# Hawkeye: an interactive visual analytics tool for genome assemblies

**DOI:** 10.1186/gb-2007-8-3-r34

**Published:** 2007-03-09

**Authors:** Michael C Schatz, Adam M Phillippy, Ben Shneiderman, Steven L Salzberg

**Affiliations:** 1 Center for Bioinformatics and Computational Biology, Biomolecular Sciences Building, University of Maryland, College Park, Maryland, 20742, USA; 2 Department of Computer Science and Human-Computer Interaction Lab, A.V. Williams Building, University of Maryland, College Park, Maryland, 20742, USA

## Abstract

Genome sequencing remains an inexact science, and genome sequences can contain significant errors if they are not carefully examined. Hawkeye is our new visual analytics tool for genome assemblies, designed to aid in identifying and correcting assembly errors. Users can analyze all levels of an assembly along with summary statistics and assembly metrics, and are guided by a ranking component towards likely mis-assemblies. Hawkeye is freely available and released as part of the open source AMOS project http://amos.sourceforge.net/hawkeye.

## Abstract 2

Hawkeye is a new, freely available visual analytics tool for genome assemblies, designed to aid in identifying and correcting assembly errors.

## Rationale

Since the DNA of the first free living organism was sequenced in 1995 [1] using the whole-genome shotgun (WGS) technique [2], hundreds of other organisms, including the human genome [3,4] and numerous model organisms, have been sequenced using WGS. The relatively low cost and high speed of the WGS method have made it the preferred method of genome sequencing for the past decade. However, achieving results of the highest quality often requires expensive manual analysis with tools that provide only a limited view of the data.

Traditional WGS projects consist of three main steps, namely sequencing, assembly, and finishing. The first stage is highly automated, whereas the latter require painstaking manual curation. In the sequencing stage, fragments of the genome are sequenced by high-throughput laboratory protocols that randomly shear the original DNA molecules into short fragments that are then sequenced. In the assembly stage, sophisticated computer algorithms operated by a human assembly team assemble these short sequences back together into a partially complete 'draft' genome sequence. Finally, in what is usually the most time-consuming stage, human 'finishers' curate the assembly to correct sequencing and assembly errors, and run additional sequencing reactions to fill in the unsequenced gaps. The result of this three-stage process is a high-quality reconstruction of the genome. However, the high cost of the finishing stage, both in terms of time and money, makes it economically unfeasible to finish any genome completely, other than relatively small ones (bacteria and viruses) and the most important model organisms (yeast, nematode, fruit fly, and human). Instead, most genomes are left in the draft stage, where some of the genome remains unsequenced and where even the assembled portions may contain significant errors.

Our primary goals are to reduce the cost of finishing genomes and to increase the quality of draft genomes by providing genome assembly teams and finishers with a visual tool to aid the identification and correction of assembly errors. In addition to these primary goals, our tool - Hawkeye 1.0 - supports numerous other analytical genome tasks, such as consensus validation of potential genes, discovery of novel plasmids, and various other quality control analyses.

Hawkeye blends the best practices from information and scientific visualization to facilitate inspection of large-scale assembly data while minimizing the time needed to detect mis-assemblies and make accurate judgments of assembly quality. Wherever possible, high-level overviews, dynamic filtering, and automated clustering are provided to focus attention and highlight anomalies in the data. Hawkeye's effectiveness has been proven in several genome projects, in which it was used to both to improve quality and to validate the correctness of complex genomes. Hawkeye can be used to inspect assemblies of all sizes and is compatible with most widely used assemblers, including Phrap [5], ARACHNE [6,7], Celera Assembler [8], AMOScmp [9], Newbler [10], and assemblies deposited in the National Center for Biotechnology Information (NCBI) Assembly Archive [11].

## Genome assembly

The need to assemble genomes has inspired many innovative algorithms that have been described in detail elsewhere [5-10,12-14]. One of the fundamental steps in any assembly algorithm is to detect how the individual sequences ('reads') overlap one another. The assembler can then use these overlaps to merge reads together, building up longer contiguous stretches ('contigs') of DNA and eventually reconstructing entire chromosomes. More than anything else, repeated sequences in the genome complicate the assembly problem beyond the ability of modern assembly algorithms, and introduce the chance of significant mis-assembly. A repetitive element can be unambiguously assembled using just overlaps only if it is spanned by an entire read. This problem motivated the development of the double-barreled shotgun sequencing approach [15], in which both ends of large fragments are sequenced, creating pairs of sequencing reads with known orientation and separation. A set of these larger fragments of similar size is called a library, and typical sizes range from 2 to 100 kilobases (kb). The end-paired reads, or mate-pairs, can be treated as a large pseudo-read with unknown interior sequence.

State-of-the-art assemblers such as ARACHNE [6,7], Celera Assembler [8], PCAP [12], Jazz [13], and Phusion [14] depend on mate-pairs to untangle false overlaps and bridge unsequenced portions of the genome to form 'scaffolds' of ordered and oriented contigs. Nevertheless, even with high quality reads and mate-pairs, repeat-induced mis-assemblies are common and range from a single incorrect base to large chromosomal rearrangements [16]. Independent validation efforts [17] and additional finishing work [18] for the intensively curated human genome sequence has identified and corrected thousands of mis-assemblies. If the human genome had been left in a draft state, future attempts to identify structural polymorphisms (for example, between human and mouse) would have been difficult if not impossible. The nature and magnitude of mis-assemblies in other genomes is largely unknown, but mis-assemblies are likely to be present in all but the most carefully scrutinized genomes.

Identifying mis-assemblies, as well as avoiding mis-assembly in the first place, is a difficult problem, mostly because of the complexity of the underlying data. The data are not only voluminous and subject to statistical variation, but also error prone because of laboratory error, machine error, and biochemical complications. Consequently, complications can occur at any level of the assembly data hierarchy (Table 1), and therefore all levels of this hierarchy must be collected and analyzed together to verify an assembly effectively. Ignoring even one level of the hierarchy can lead to false assumptions, just as an assembler that ignores mate-pair evidence risks mis-assembly in repetitive regions. Hawkeye is the first analysis tool that enables users to navigate the assembly hierarchy easily, and thus enables a complete and accurate analysis of the assembly.

**Table 1:** Hierarchy of assembly data types

Data type	Description
Scaffold (100 kb to 10 Mb)	Layout of potentially nonoverlapping contigs based on mate-pair information, ideally spanning entire chromosomes or replicons
Contig (5 kb to 500 kb)	Layout of overlapping reads with a consensus sequence
Mate-pair (2 kb to 100 kb)	Pair of end-sequenced reads with a known orientation and separation
Read (0.5 kb to 1.0 kb)	Base-calls and quality scores assigned to a chromatogram
Chromatogram (4× 10,000 time points)	Signal data from a sequencing reaction of a physical piece of DNA

Each type is composed of the next lower level type. Typical sizes are also listed. bp, base pairs; Mb, megabases.

### Assembly visualization and analysis

Prior work on genome assembly visualization has focused on three different levels of assembly artifacts. The first focuses on the raw signals emitted by sequencing machines as exemplified by the four-color chromatograms displayed at the NCBI Trace Archive [19]. The second is visualization by tools such as Consed [20], which focus on the overlaps and alignment of reads within contigs and allow for detailed inspection of the consensus sequence and its support. The third highlights the mate-pair relationships either between or within contigs, and is commonly displayed as linked arrows or line segments as in the NCBI Assembly Archive [11].

Mate-pair visualization most directly addresses the validation of an assembly by highlighting discrepancies between expected and observed read placements. Clusters of mated reads that are statistically too close together or too far apart are signatures of deletion and insertion mis-assemblies, whereas occurrences of mis-oriented mate-pairs, or reads whose mate-pair are missing, are indicative of other types of mis-assembly. Tools such as Celamy [21], BACCardI [22], and the clone-middle diagrams proposed by Huson and coworkers [23] effectively highlight these 'unhappy' mates. TAMPA extends this idea further, and provides a positional bound for the mis-assembly event [24].

After a genome is sequenced and assembled, various meta-data, such as gene predictions, are computed and attached to particular intervals on the sequence. Genome browsers such as Ensembl [25], GBrowse [26], CGView [27], and the UCSC Genome Browser [28], lay the features out on either a linear or circular coordinate system as a set of arrows. Additional continuous information, such as GC content or alignment similarity, is often plotted as well. This type of view is widely popular among biologists because it brings multiple sources of evidence into a single display and can be made available over the web. However, these tools are poorly suited for assembly visualization because they cannot capture underlying sequence and assembly data, in part because of the large datasets involved.

In addition to visualizations, various statistics have been described for the validation of read layouts. The A-statistic [8] compares the distribution of individual reads against a statistical model of random read coverage to detect contigs whose coverage is too deep, suggesting a collapsed repeat. Another measure, the Compression-Expansion (CE) statistic [29], developed by Roberts and coworkers at the University of Maryland IPST Genome Assembly Group, quantifies the degree of compression or expansion for the set of mate-pairs spanning any particular position in the assembly. It is computed on a per library basis as the mean of the insert sizes spanning a position minus the mean value of the library divided by the standard error (the library standard deviation multiplied by the square root of the number of inserts at the position). The expected value of the CE statistic is zero, which occurs when inserts spanning a position have a size distribution that matches the global library distribution. CE values far from 0 outside the interval [-3, +3] indicate an unexpected distribution of insert sizes at that location. Certain mis-assemblies, such as collapsed repeats, generate characteristic insert size distributions with large negative CE values, whereas insertion mis-assemblies produce large positive CE values.

## The Hawkeye interface

### Launch Pad

Effective overview, ranking, and navigation components are the keys to exploring large data spaces, just as sightseeing is more effective with a map, tour guide, and car. The Hawkeye Launch Pad is the first view presented to the user and it is designed to address these three needs as well as answer the first questions any analyst has about an assembly: 'How big are the contigs?' and 'How good is it?'

**Figure 1:**
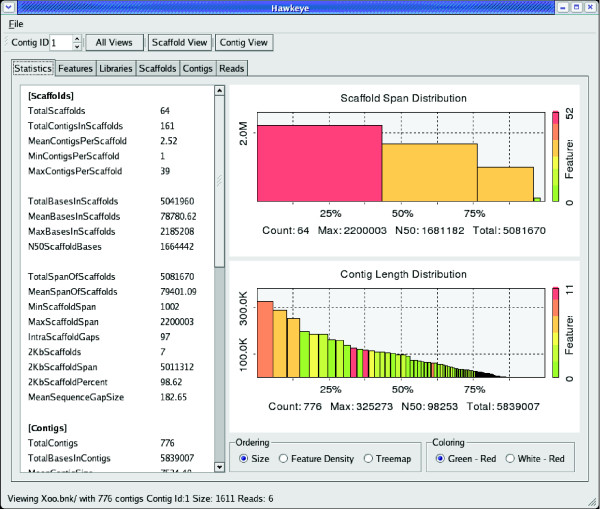
The Hawkeye Launch Pad. Scaffolds and Contigs are plotted so that the size of the scaffold represents the size of the object. The color of the rectangle indicates the number of mis-assembly features. Details and other abstract visualizations are available through the tabbed interface.

To answer these initial questions graphically, Launch Pad displays two N-plots in its initial view: one for contigs and another for scaffolds. An N-plot is a bar graph based on the popular N50 assembly metric (Figure 1). Each bar represents a contig (or scaffold), where the height of the bar represents its length in base pairs and the width represents its length as a percentage of the genome size. This plot gives immediate feedback on both the size and number of contigs contained within the assembly. A few wide steps covering most of the x-axis indicates that the assembly contains a small number of large contigs, whereas many steps of the same size indicate a fragmented assembly. In addition to N-plots, contig and scaffold sizes also can be visualized as a space-filling Treemap [30]. Various other assembly statistics are presented in text-based tables for detailed inspection of high-level assembly quality.

Seo and Shneiderman [31] advocate a generalized rank-by-feature framework for the exploration of multivariate data sets to guide exploration and expedite the discovery process. Hawkeye employs a ranking strategy for contigs and scaffolds that was inspired by the rank-by-feature framework. The first ranking criterion is size, which is implicit in the N-plot described above. The second ranking criterion focuses on contig or scaffold quality, and is encoded in the N-plot by color. Contigs and scaffolds with a high density of mis-assembly signatures (those likely to be mis-assembled) are shaded red in the N-plot, whereas contigs and scaffolds with a low density (those less likely to be mis-assembled) are shaded green. Mis-assembly signatures are regions in the assembly with characteristics indicative of a mis-assembly, such as a cluster of compressed mate-pairs, which suggests a collapsed repeat. Utilities bundled with the software pre-compute some useful mis-assembly indicators such as read polymorphism, alignment breakpoints, and regions with poor insert 'happiness', although users can easily load new metrics via an XML-like interface as additional assembly metrics are invented. Short descriptions of the included metrics are given below in the discussion of the interface components.

Ranking scaffolds and contigs by size and feature density guides users directly to the regions that require the most attention. This minimizes the time needed to pinpoint potential trouble, and provides the ability to drill down to either the scaffold or contig level to examine interesting objects and features in greater detail. Users simply double click in the N-plot to display a new window with the selected contig or scaffold in the more detailed scaffold or contigs views described below. In addition, users can click on other tabs in the Launch Pad to display sortable tables of scaffold, contig, read, library, and feature information. Histograms of insert sizes, GC content, and other attributes are also available that permit quality inspection of other aspects of the assembly.

### Scaffold View

**Figure 2:**
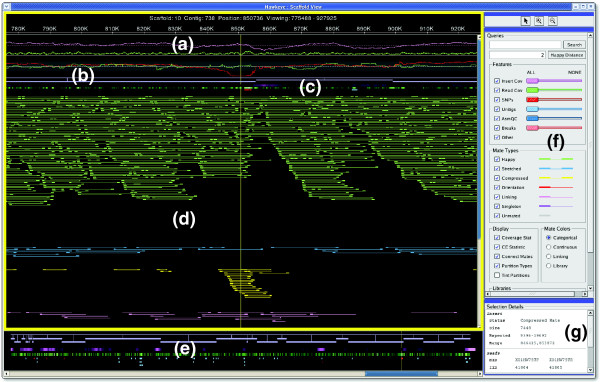
The Hawkeye Scaffold View. The scaffold view displays the insert panel, outlined with a yellow border, consisting of **(a) **plots of statistical information, **(b) **scaffolded contigs, **(c) **feature tracks, and **(d) **inserts. Also displayed are the **(e) **overview panel, **(f) **control panel, and **(g) **details panel. The insert panel displays the details and individual inserts for regions of the scaffold selected in the overview panel, whereas unselected regions are grayed out in the overview. By default, inserts are colored by category (green→happy, blue→stretched, yellow→compressed, purple→singleton). The eye is drawn to the cluster of compressed mates towards the bottom of the insert panel.

The Scaffold View provides an abstract graphical view of the assembly, and is often the most natural view to pursue after identifying an item of interest in the Launch Pad. This view displays the read layout on a per scaffold basis, along with integrated assembly statistics and feature information. The view consists of three panels: the Overview Panel, the Insert Panel, and the Control Panel (Figure 2).

The Overview Panel (Figure 2e) displays the entire current scaffold as a linear ordering of connected contigs along the x-axis, with the assembly features displayed below. The width of the contig boxes and the gaps between them are proportional to the length and separation of contigs, respectively, and contigs are 'scaffolded' together by conjoining lines. Assembly features are laid out below the contigs in multiple tracks. The first two tracks are heat map plots of insert and read depth of coverage that color code coverage regions significantly above or below the mean value. Positions in the assembly with a coverage level near the mean are shaded to blend with the background, whereas positions significantly deviating from the mean, such as in collapsed repeats, are given a contrasting color to the background. Interval features are displayed in additional tracks below the coverage tracks. These discrete features are preloaded with the assembly data and represent arbitrary regions of interest, such as regions with mis-assembly signatures, or sequence characteristics such as gene models, and so on. Large features or clusters of different feature types demand attention and take precedence over small, isolated features. All feature tracks can be filtered by value (score or size), allowing users to focus their attention on the most egregious or interesting features.

The Insert Panel (Figure 2d) provides a detailed look of the region selected in the Overview Panel. Users select regions to investigate in the Insert Panel with a magnifying glass tool, or by adjusting the scroll bars beneath the overview. At the top of the Insert Panel, statistical line plots (Figure 2a) display the depth of read (green) and insert coverage (purple) along with the CE statistic value for each library along the scaffold. The coverage tracks will vary from 0 to the maximum depth of coverage, but the CE statistic track is fixed to display values in the range [-6,6] because the CE statistic value will be near 0 except in mis-assembled regions. Users can read the precise coverage or CE values by clicking on the plot that displays the value in the details panel. Extreme values or variation in any of the statistical tracks can indicate mis-assembly or other assembly issues and encourages users to look at statistically anomalous regions more thoroughly.

A plot of the depth of k-mer coverage is optionally plotted overlaying the read and insert coverage. It displays the number of occurrences in the set of reads, of the substring of length k starting at each position along the contig consensus sequences. K-mer coverage spikes reveal the repeat structure of the genome and highlights regions of potential mis-assembly. Correctly assembled unique sequence has k-mer coverage approximately equal to the read coverage, whereas repeat sequences have k-mer coverage that is a function of the number of copies of the repeat, regardless of whether the repeat has been correctly assembled.

**Figure 3:**
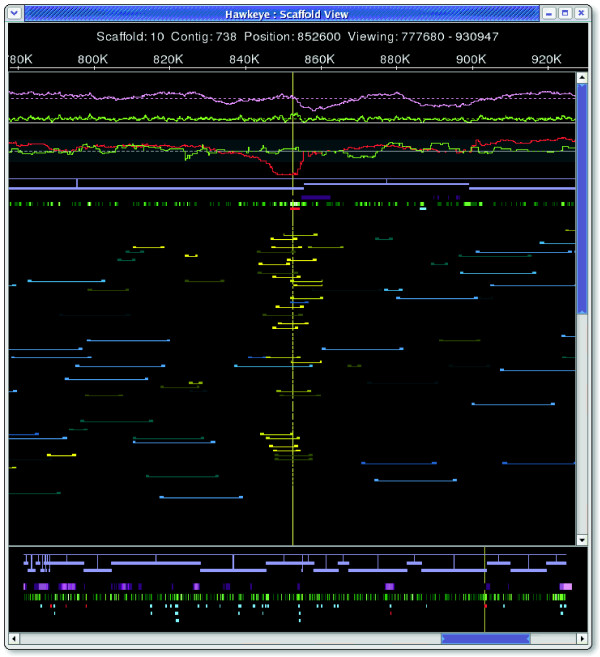
Mis-assembly detection in Scaffold View. Continuous coloring in the Scaffold View displaying a region of *Xanthamonas oryzæ*. Slightly compressed mate-pairs are colored increasingly bright yellow as they deviate from the mean. Slightly expanded pairs are also visible in blue, but are uncorrelated and most likely caused by inexact library sizing.

Below the contig and feature tracks lies the layout of the sequencing reads (Figure 3d). The reads are drawn as colored boxes connected to their mate by a thin line. If it is not possible to connect a read with its mate because of misplacement or other issues, a thin line is drawn proportional to the expected size of the insert. Using a size threshold based on the standard deviation of the library (called 'happiness' within the interface), and the orientation constraints of the mate-pair relationship, inserts are categorically grouped to enhance visibility and emphasize clusters of unexpected sizing or inconsistent mate-pair orientation (Table 2). Unfortunately, subtle mis-assemblies can be overlooked if most of the mis-assembled inserts fall within the happiness threshold, and so an alternative continuous coloring scheme is available. In this scheme, happy inserts are shaded into the background to make them less visible, while stretched and compressed mates are given brighter colors corresponding to how compressed or expanded they are. Positions spanned by inserts that are even slightly skewed will show as clusters of bright, similarly colored inserts, indicating a possible problem (Figure 3). This view is more sensitive than setting arbitrary thresholds and has proven to be quite effective for identifying mis-assemblies missed by categorical analysis.

**Table 2:** Categorization of insert happiness

Insert Type	Description	Color
Happy	Correctly oriented and sized	Green
Stretched	Correctly oriented, but larger than expected	Blue
Compressed	Correctly oriented, but smaller than expected	Yellow
Mis-oriented	Mates point away or in same direction	Red
Linking	Mates are in different scaffolds	Pink
Singleton	The read's mate is unplaced	Purple
Unmated	No mate associated with read	Grey

Inserts with size violations (stretched or compressed) are reported with respect to a user configurable parameter for the maximum acceptable number of standard deviations from the library mean for that insert (default 2).

The coordination of multiple forms of evidence combined with user interaction is the key to the Scaffold View's effectiveness. Statistical spikes, feature clusters and contrasting insert colors combine to guide users to the important areas of the assembly. However, the underlying DNA sequences and chromatogram traces are absent from this view, and so another level of detail is required. This is handled by the Contig View, which is essentially a vertical slice of the Scaffold View displaying the read tiling in full detail with base-calls and chromatogram traces. The two views are synchronized, so that a user click in the background of the Insert Panel centers the Contig View to that position.

### Contig View

**Figure 4:**
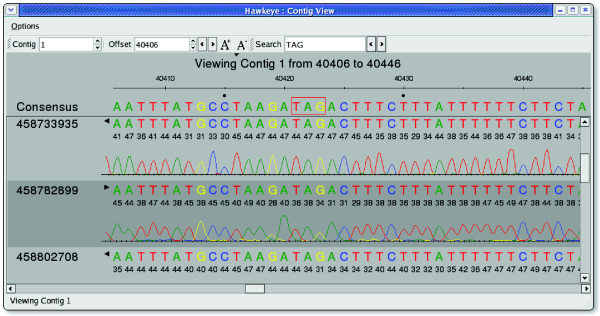
The Hawkeye Contig View. Quality values and chromatograms are displayed on demand in the Contig View to confirm a potential stop codon outlined in red in the consensus.

Similar to the Scaffold View, the Contig View also displays the read tiling, except the abstract rectangles from the Scaffold View are replaced with the actual strings of base-calls for each read (Figure 4). The reads supporting the consensus at each position are arranged so that their individual bases are aligned vertically, including gaps inserted by the assembler to maintain the alignment. Consensus positions in which the underlying reads disagree are marked, and dissenting base-calls are highlighted.

The Contig View can also display base-call quality values and chromatogram traces (if available) to examine discrepancies in more detail. Quality values are loaded with the assembly data, and the traces are either loaded from the file system or downloaded on-the-fly directly from NCBI Trace Archive or other archives. In the Contig View, the chromatograms may be compressed or expanded to ensure consistency between the reads, but double-clicking on a read displays the undistorted chromatogram for the selected read in a new window. Human examination of the trace data is often necessary to confirm conflicting base-calls as sequencing error or genuine single nucleotide polymorphisms (SNPs). False SNPs caused by sequencing or base-calling errors are quite common and can be largely ignored, whereas SNPs supported by the chromatogram or occurring in multiple reads at the same position must be examined more closely.

**Figure 5:**
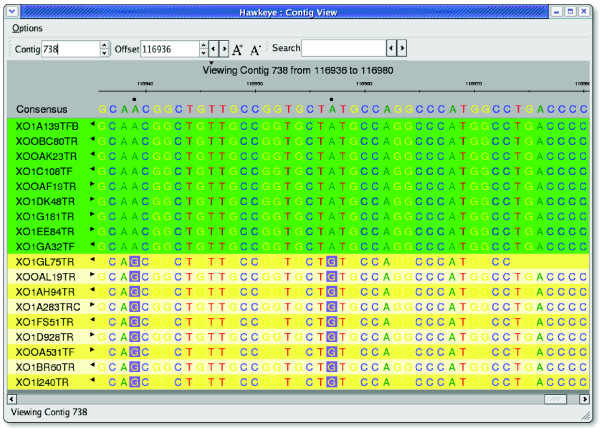
SNP sorted reads in the Contig View. Clicking in the consensus automatically clusters the reads into correlated groups by sorting and coloring the reads by their base at that position. SNP, single nucleotide polymorphism.

When two or more reads share a discrepancy from the multi-alignment, we call this a correlated SNP. Because most SNPs are caused by random sequencing error, it is highly unlikely that a random error in two separate experiments will occur at exactly the same position, especially if those bases have high quality values. Although biological or biochemical explanations can sometimes account for this correlated error, it is commonly caused by mis-placed reads from different positions in the genome, especially for haploid organisms. One very common cause of a correlated SNP is the collapse of two near-identical copies of a repeat into a single copy by the assembler. Because both copies of the repeat should have been sampled evenly, the same number of reads should be present for each copy, and the reads will partition into two equally sized groups distinguished by the differences in the multiple alignment. In addition to flagging these regions in the Scaffold View, the Contig View supports the separation of these groups via on-the-fly clustering of correlated discrepancies. Clicking the consensus base in question sorts the underlying reads into groups based on the base-calls at that position (Figure 5).

**Figure 6:**
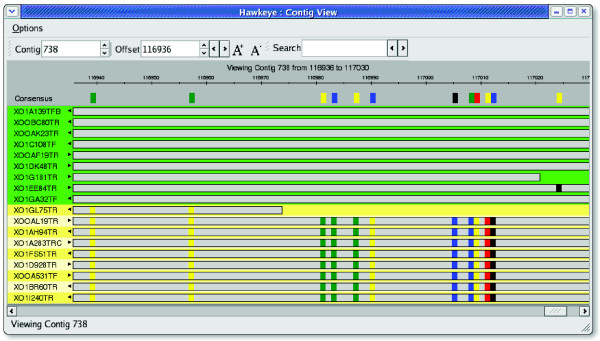
Semantic zooming in the Contig View. Semantic zooming shifts from displaying the individual base pairs in reads to a compact abstract SNP-Barcode in which only bases that disagree with the consensus are colored thus displaying a wider range of a contig. SNP, single nucleotide polymorphism.

In addition to SNPs correlated by row, they also can be correlated across multiple columns of the multi-alignment. In this case, it can be difficult to fit all the correlated columns on the screen at once, and so the Contig View employs a semantic zooming mechanism for viewing large regions of the multi-alignment simultaneously. Zooming out reduces the size of the base-calls until the text becomes unreadable. At this point, the view switches to a 'SNP barcode' view, inspired by the software DNPTrapper [32]. In this view, agreeing bases are blended with the background to remove them from view, and only the disagreeing bases are colored (Figure 6). Reads that share the same pattern of SNPs are quickly identified and can be clustered together as before.

## Results

We designed Hawkeye to enhance understanding of genome assemblies and to assist in the detection and correction of assembly errors. Below we outline a sample of analysis tasks possible with Hawkeye.

## Assembly validation

We applied Hawkeye to inspect potential mis-assemblies systematically in the draft assembly of a recent genome sequencing project for the bacterium *Xanthamonas oryzæ *pv.*oryzicola *[33]. The 4.8 megabase (Mb) genome was sequenced in 62,229 end-paired shotgun reads representing approximately 9× coverage of the genome. The reads were assembled with Celera Assembler using default parameters. Over 96% of the assembly was contained in three large scaffolds, each over 1 Mb in size. Hawkeye uncovered a number of mis-assemblies that were present in the draft assembly.

One mis-assembly was discovered near the end of a contig in the third largest scaffold. The evidence for the mis-assembly was threefold: elevated read coverage, the presence of compressed mate-pairs, and correlated SNPs within the reads. As explained above, this combination of evidence suggests that the reads from two or more instances of a repeat have been collapsed into a single instance.

The Scaffold View has strong support for the hypothesis of a collapse. It includes a spike in read coverage in this region, to more than twice the mean (Figure 3). In the default categorical view, only one mate-pair is classified as compressed using a threshold of three standard deviations from the mean. However, the continuous insert coloring reveals a cluster of moderately compressed mates in this region (colored yellow). Furthermore, clicking in the CE statistic plot shows the CE statistic for this region falls to -6.36, which is well below the threshold of -3.0 for likely compression type mis-assembly. Finally, the red features spanning the area indicate a high level of read polymorphism. The coordinated Contig View shows two distinct clusters of reads, probably representing the two repeat copies that were collapsed together (Figures 5 and 6).

Following our discovery, we created a second assembly using just the reads and mates from the collapsed region with stricter parameters for the assembler, which required a greater degree of similarity between overlapping reads. This local assembly was inspected, and did not have any mis-assembly signatures. A contig alignment dot plot generated by Nucmer [34] revealed that the collapsed repeat did not occur exactly in tandem, but contained an additional approximately 500 base pairs of unique sequence between the two repeat copies that was missing from the original assembly. The mis-assembled region was replaced with the corrected local assembly using the AMOS tool stitchContigs [35], providing an accurate consensus sequence for gene annotation.

### Assembly diagnostics

Hawkeye also has proved useful for improving assemblies globally by explaining why assemblies are worse than expected. The initial assembly for the *Bacillus megaterium *sequencing project (Ravel J, personal communication) had a surprisingly large number of small scaffolds given the expected read and insert coverage levels. The genome size was estimated at about 5 Mb, and the 74,000 shotgun reads should have provided 12× read coverage and nearly 50× insert coverage of the genome. Despite adequate sequencing, the assembly had on average less than 10× read coverage and no scaffold larger than 1 Mb. Furthermore, over 12% of the reads were left out of the assembly (called 'singletons').

We explored the source of the fractured assembly by inspecting the largest scaffold. We quickly discovered a high percentage of singleton mates (reads in the scaffold whose mates were singletons). Clusters of singleton mates can be caused by deletion mis-assemblies, but the singleton mates in this assembly were distributed evenly throughout the scaffold, and were not correlated with other mis-assembly features. Another likely cause of singleton mates is low read quality, below what the assembler will tolerate. For example, with default parameters, Celera Assembler will not assemble together reads if they disagree by more than 1.5%. To test for low read quality, we examined the largest contig using Hawkeye's SNP barcode view with a quality value heat map. As suspected, the ends of the reads were lower quality than the interior, but we were surprised to find clusters of differences near the ends of individual reads. Furthermore, these differences were not correlated and all were deletion events.

This combination of evidence suggested that the base-caller systematically missed peaks near the ends of chromatograms. These missed peaks fell in relatively low quality regions, so we re-trimmed the reads with more aggressive parameters, and re-assembled the genome. This re-trimming reduced the number of singleton reads to fewer than 2% and greatly improved scaffold and contig sizes. In a follow-up investigation, we discovered that the base-calling software in the sequencing pipeline had been updated recently, but the trimming software had not been appropriately recalibrated.

### Discovery of novel plasmids

The assembly of *Bacillus megaterium *also was interesting because the organism was thought to have seven plasmids in addition to the main chromosome of the organism. The complete sequence for four plasmids was previously available, but the sequences for the others were not. After assembly, we inspected the scaffolds using Hawkeye to find the novel plasmids by searching for circular scaffolds. In a linear version of a circular scaffold, reads near each end of the scaffold will be oriented such that their mate would fall outside the scaffold, while instead those mates will appear within the scaffold at the opposite end. In addition, these mates will appear in Hawkeye as mis-oriented mates occurring on the ends of the scaffold without the presence of other mis-assembly evidence. We identified seven scaffolds with this structure, and four matched the known plasmid sequence. The additional circular scaffolds are the three novel plasmids (laboratory confirmation is pending).

### Consensus validation

During the genome sequencing and annotation of the 160 Mb parasite *Trichomonas vaginalis *[36,37] a large number of 'split genes' were identified. In a split gene, two adjacent open reading frames (ORFs) are separated by a stop codon, but in other organisms' homologous genes the entire region is a single ORF forming a single functional gene.

We attempted to confirm the correctness of these split genes by ruling out the possibility of mis-assembly and confirming the accuracy of the consensus sequence. The split gene annotations were loaded as features into Hawkeye. We then systematically checked for potential mis-assemblies near these genes in the Scaffold View, but found only happy inserts and no evidence of mis-assembly. In the Contig View, we examined the chromatograms and quality values for base-calls in these regions, looking particularly for mis-calls that would have introduced frame shifts or false stop codons. After finding no consensus discrepancies or signs of mis-assembly, we concluded the sequence was correct, and the genes had not been mis-assembled. The reads in this region came from several different genomic libraries, providing further evidence that the split genes are not an artifact of library construction.

## Discussion

Cognitive psychologist and computer science researcher Herbert Simon stated, 'Solving a problem simply means representing it so that the solution is obvious' [38]. In this spirit, Hawkeye strives to provide a visual, manipulable interface to help finishers understand and reason about complex assembly data. In addition to providing a useful interface for the examination of assembly data, Hawkeye further supports the analytical process by providing statistical and computational data analysis, enabling users both to reduce data complexity and to form accurate judgments.

Hawkeye addresses the issues of scale and complexity by guiding users to the most likely areas of mis-assembly, and adhering to the visual information seeking mantra: overview first, zoom and filter, then details-on-demand [39]. The main application window, or 'Launch Pad', acts as a global overview by displaying summary assembly statistics, along with graphs and sortable tables of assembly information. The ranking component of this display encourages users to inspect regions of the assembly in order of importance: largest to smallest and low quality to high quality. The more detailed 'Scaffold View' is capable of displaying an entire contig or scaffold and its underlying reads on a single screen for scaffolds spanning 10+ Mb of sequence and 100,000+ reads. Alternatively, users can zoom in and filter the display to focus on particular regions of interest. Finally, the lowest level assembly information is displayed in the coordinated 'Contig View', displaying the consensus sequence, read-tiling, base-calls, and supporting data. Coordination among these three views - Launch Pad, Scaffold View, and Contig View - allows for very efficient top-down analysis of even the largest assemblies. It leads the user to a natural analytic progression: discern high-level quality from statistics and features; examine a poorly scoring scaffold for mis-assembly at the clone-insert level, looking for uneven insert distribution and improperly sized or mis-oriented mate-pairs; examine possible mis-assemblies in more detail at the base-call and chromatogram level, looking for correlated discrepancies supported by chromatogram traces; and confirm or refute hypothesis of mis-assembly.

After confirming the presence of mis-assemblies, users have a choice of methods for correcting the assembly. If there are numerous or systematic errors, the best solution is often to reassemble the genome after adjusting the assembler parameters, such as adjusting the read trimming to be more conservative, or requiring a higher degree of similarity between overlapping reads to correct for collapsed repeats. If the errors are more localized, such as collapsed repeats or mis-placed reads, users can correct the individual mis-assemblies with the companion AMOS tools [35] or with other third party tools. Other assembly complications, such as high levels of sequencing error, can be automatically corrected with tools such as AutoEditor [40].

Hawkeye combines computational predictors with interactive visualizations to enable efficient and accurate human inspection of assembly data, resulting in decreased verification costs and higher quality data for the scientific community. We have utilized its ranking component to detect the presence of localized mis-assemblies in various genome assemblies, and have used its abilities to verify the correctness of reassemblies. We have also used it to improve genome assemblies globally by identifying systematic problems with read trimming, which had fragmenting assemblies. Finally, we have positively identified biologically interesting phenomena such as novel plasmid sequences, and demonstrated how Hawkeye can be used to confirm the base-call level consensus sequence of contigs to verify the accuracy of unusual gene structure.

Hawkeye 1.0 emphasizes visual presentation, but future versions should include capability to edit individual bases, manipulate contigs, and interactively mark regions for further attention. We also plan to improve visualizations for new sequencing technologies such as the display of flowgrams used in 454 sequencing. Finally, we also plan to improve support for gene annotation tasks, including displaying the translated amino acid sequence in addition to the DNA sequence and enhanced support for displaying gene models with introns.

Hawkeye is a desktop GUI application written in C++, and requires the Qt graphics library, which is freely available from Trolltech [41]. Otherwise, users can load and analyze assemblies without any other dependencies on Linux/Unix, Microsoft Windows (with Cygwin), and Mac OS X based computers. Desktop machines with 1 GB of RAM will easily accommodate small to mid-sized assemblies (<200,000 reads), whereas more RAM may be necessary for larger assemblies to remain responsive. The user manual and source code for Hawkeye are available from the Hawkeye website [42].
